# Supplementary material for: Utilization Pattern of Acupuncture and Its Associated Predictors for Cancer Pain in South Korea: A Cross-Sectional Study
Source: Diseases. 2025 Sep 2;13(9):292. doi: 10.3390/diseases13090292 (PMC12468660; doi:10.3390/diseases13090292)
Supplement: Supplementary file 1 [file diseases-13-00292-s001.zip › diseases-3813951-supplementary.pdf]

Survey Questionnaire

Integrative Medicine Survey for Pain Management: Understanding the Experiences, Needs, and Barriers of Cancer Patients

Principal investigator	Hwaseung Yoo
Study ID	MSK_DJU_SR

Participants identification code	Participants initials
**MSK_DJU_SR** ☐☐☐	☐☐☐

Section I. 인구학적조사 Demographics

1. 생년월일 (DOB)-------------------------------------- ☐☐☐☐ 년 ☐☐ 월 ☐☐ 일

2. 병록번호 (Patient number)-- ☐☐☐☐☐☐

3. 성별 (sex) -- ◯ 남성 (M) ◯ 여성 (F)

4. 키 / 몸무게 (Height/Weight)------------------------ ☐☐☐ . ☐☐ CM ☐☐ . ☐ kg

5. 인종 (Race) ----------------------- ◯ 한국인 (Korean) ◯ 외국인 Foreigner (____________)

6. 민족성 (Ethnicity)------------------------- ◯ 아시안 (Asian) ◯ 기타 Others (__________)

7. 교육 (Education)

◯ 중학교 졸업 이하 (elementary school – 11 Grade)

◯ 고등학교 졸업 (High school diploma)

◯ 4 년제 대학 (4 years college degree)

◯ 대학원이상 (Graduate or professional Degree)

8. 고용형태 (Employment)

◯ 풀타임 Full time (주 30 시간 이상 ≥ 30 hours/ wk)

◯ 파트타임 Part time (주 30 시간 미만 / < 30hrs/ wk)

◯ 고용되지않음 not currently employed

9. 가구조사 Living arrangement

◯ 혼자 거주 Living Alone

◯ 다른 성인과 거주 Living with other adults

◯ 미성년자/ 미성년자 들과 거주 (성인 없음) Living with child/ children but no adults

10. 소득수준 (연간) Annual Income

◯ 3000 만원 이하 (3million KRW) ◯ 3000 – 5000 만원 (3-5 million KRW)

◯ 5000-7000 만원(5-7million KRW) ◯ 7000 만원 이상 (more than 7million KRW)

11. 어떤 종류의 암으로 치료, 또는 관리를 받고 계시나요?

What type of cancer are you receiving treatment or follow up care?

◯ 유방암 Breast Cancer ◯ 폐암 Lung Cancer ◯ 전립선암 Prostate cancer

◯ 직장/대장암 Colon/Rectal Cancer ◯ 두경부암 Head/ Neck Cancer

◯ 피부암 Melanoma ◯ 백혈병 leukemia ◯ 위암 Gastric Cancer

◯ 췌장암 Pancreas Cancer ◯ 간암 Liver Cancer ◯ 기타 Other: _______________

12. 암 치료 후 관리를 위해서 방문하셨나요?

Are you here for a post-treatment follow up visit with your oncologist?

◯ 네, 저는 수술, 항암치료, 방사선치료 같은 치료를 마쳤습니다. Yes, I have completed active cancer treatments such as surgery, chemotherapy, or radiotherapy.

◯ 아니요, 저는 아직 수술 항암 방사선 등의 치료를 받고 있습니다. No, I am currently receiving active cancer treatments.

◯ 아니요, 저는 최근에 암을 진단받아 곧 치료를 시작할 예정입니다. No, I have been recently diagnosed and am about and am about to start cancer treatments.

◯ 기타 (설명 부탁 드립니다)______________ other: specify

13. 암 진단을 받은 후 어떤 치료를 받으셨나요? (해당사항을 모두 체크해주세요)

Since your cancer diagnosis, have you had the following cancer treatments? (Check all that apply)

◯ 수술 Surgery ◯ 항암치료 Chemotherapy ◯ 방사선치료 Radiation Therapy

◯ 재건수술 Reconstructive Surgery ◯ 면역치료 Immunotherapy/Biological therapy

◯ 골수이식 Bone marrow transplant ◯ 호르몬치료 Hormonal Therapy

◯ 기타 Others :____________________________________

Section II. 통증 Pain

많은 암환자들은 종종 통증을 경험하곤 합니다. Many patients with your illness experience pain from time to time.

1. 귀하의 통증이 **지난 24 시간** 동안 **가장 심했을 때,** 그 정도를 가장 잘 나타내는 숫자에 동그라미를 하십시오.

   Please rate your pain by filling the one number that best describes your pain at its WORST in the past 24 hours.

0	1	2	3	4	5	6	7	8	9	10
통증 없음										상상할 수 없을 정도로 심한 통증

2. 귀하의 통증이 **지난 24 시간** 동안 **가장 약했을 때**, 그 정도를 가장 잘 나타내는 숫자에 동그라미를 하십시오.

   Please rate your pain by filling the one number that best describes your pain at its LEAST in the past 24 hours.

0	1	2	3	4	5	6	7	8	9	10
통증 없음										상상할 수 없을 정도로 심한 통증

3. 귀하가 느끼는 통증의 **평균 정도를** 가장 잘 나타내는 숫자에 동그라미를 하십시오.

   Please rate your pain by filling the one number that best describes your pain on AVERAGE.

0	1	2	3	4	5	6	7	8	9	10
통증 없음										상상할 수 없을 정도로 심한 통증

4. 귀하가 **바로 지금** 느끼는 통증의 정도를 가장 잘 나타내는 숫자에 동그라미를 하십시오.
   Please rate your pain by filling the one number that best describe your pain RIGHT NOW.

0	1	2	3	4	5	6	7	8	9	10
통증 없음										상상할 수 없을 정도로 심한 통증

5. 지난 24 시간 동안 통증이 귀하에게 얼마나 지장을 주었는지 가장 잘 나타내는 숫자에 동그라미를 하십시오.
   Please fill the number that best describes how, during the past 24 hours, pain has interfered with your

가. 전반적인 활동 General activity

0	1	2	3	4	5	6	7	8	9	10
지장을 주지 않음										완전히 지장을 줌

나. 기분 mood

0	1	2	3	4	5	6	7	8	9	10
지장을 주지 않음										완전히 지장을 줌

다. 보행능력 walking activity

0	1	2	3	4	5	6	7	8	9	10
지장을 주지 않음										완전히 지장을 줌

라. 통상적인 일 (집안, 밖의 일 다 포함)normal work (work outside, housework)

0	1	2	3	4	5	6	7	8	9	10
지장을 주지 않음										완전히 지장을 줌

마. 대인관계 Relationship with other people

0	1	2	3	4	5	6	7	8	9	10
지장을 주지 않음										완전히 지장을 줌

바. 수면 Sleep

0	1	2	3	4	5	6	7	8	9	10
지장을 주지 않음										완전히 지장을 줌

사. 인생을 즐김 enjoyment of life

0	1	2	3	4	5	6	7	8	9	10
지장을 주지 않음										완전히 지장을 줌

Section Ⅲ. 통증관리 Pain Management

1. 암 진단을 받기 전에, 본인을 만성통증 환자라고 생각하셨습니까**? Before your cancer diagnosis,** did you consider yourself to be a person who lived with chronic pain?

   ◯ 네 YES ◯ 아니요 NO

2. 암 진단을 받은 후, 몇몇 암 치료법은 일부 사람에게 통증을 일으킬 수 있습니다. 지난 7 일 동안 다음과 같은 증상을 어느 정도 경험하셨나요? **Since your cancer diagnosis:** Some cancer treatments may cause pain in some people but not in others. To what extent have you experienced the following symptoms in the **past 7 days**?

	없음	약함	중간	심함
a. 수술부위의 통증 Pain at site of surgery	◯	◯	◯	◯
b. 방사선 치료와 관련된 통증 Pain at site of radiation	◯	◯	◯	◯
c. 신경병증 (저림, 감각이상, 화끈거림) Neuropathy (tingling, burning, or numbness in hands or feet)	◯	◯	◯	◯
d. 항암 치료 후 근골격계 통증 Muscle ache and pain following chemotherapy	◯	◯	◯	◯
e. 기타, 설명 부탁 드립니다. Other, please specify:-	◯	◯	◯	◯

____

____

____

3. 지난 3 개월을 돌아보았을 때, 스스로를 만성통증 환자라고 생각하셨나요? **During the**

past 3 months, do you consider yourself to be a person who lives with chronic pain?

◯ 네 ◯ 아니요

4. 지난 7 일 동안 통증관련 약을 드셨나요? (해당항목 모두 선택해주세요) Have you taken any pain medications in the **past 7 days**? (*CHECK ALL THAT APPLY*)

◯ 비스테로이드 소염제 NSAIDS (e.g. Motrin/Ibuprofen, Aleve/Naproxen, Celebrex/Celecoxib)

◯ 타이레놀 Tylenol (Acetaminophen)

◯ 신경병증치료제 Nerve pain pills (e.g. Neurontin/Gabapentin, Pregabalin, Lyrica)

◯ 항우울증약 Anti-depressants (e.g. Cymbalta/Duloxetine, Effexor/Venlafaxine, Elavil/Amitriptyline)

◯ 근육이완제 Muscle relaxants (e.g. Flexeril/Cyclobenzaprine, Baclofen, Skelaxin/Metaxalone)

◯ 마약성 진통제/오피오이드 Narcotics/Opioids (e.g. Percocet/oxycodone, Dilaudid/hydromorphone, Fentanyl, Morphine)

◯ 기타, 설명 부탁 드립니다. Others, please specify ___________________________

◯ 통증관련 약을 먹고 있지 않습니다. I am not taking any pain medications.

5. 아래 항목 중 통증조절을 위해 이용해본 것들을 선택해주세요 (해당사항 모두 선택) Have you used any of the treatments listed below to manage your pain? (Check all that apply)
   a. 침 치료 Acupuncture
   b. 한약치료 Herbal medicine treatments
      i. 통증을 조절하기 위해 어떤 종류의 한약을 사용해 보셨나요? Which herbs do you use or have you used in the past for managing your pain? ______________________________
   c. 침, 한약치료를 해본 적 없다. None of the above

6. 일반적으로 의사, 한의사, 간호사에게 제공받은 통증치료에 대해 만족하십니까? In general, are you satisfied with how your doctors and nurses manage your pain?

◯ 네 Yes

◯ 아니요: (아래칸에 불만족스러운 부분에 대한 설명 부탁 드립니다)

No: Please describe below **why** you are dissatisfied:

____

____

____

통증관리에 대한 자세와 믿음
Attitudes and Beliefs about Pain Management:

7. 모든 사람은 각자의 통증조절에 방법에 있어서 다른 믿음을 가지고 있습니다. 이에 따라 다른 통증조절 방법을 선택하곤 합니다. 아래 질문에 대하여 **어느 정도 동의, 또는 비동의** 하시는지 선택 부탁드립니다.

Each individual may have different beliefs that help him/her choose different types of **pain medication.** Please select the best option to indicate the extent to which you agree or disagree with the following statements.

질문	비동의	약한비동의	모르겠다	약한동의	강한동의
**a. 통증관리를 위한 치료들은 부작용이 없어야 한다.** Pain management treatments should have no negative side effects.					
**b. 통증관리를 위한 치료는 독성이 없어야 한다.** It is important to me that pain management treatments are non-toxic.					
**c. 통증관련 치료는 자연적 요소들로 만 이루어져야 한다.** Pain management treatments should only use natural ingredients					
**d. 통증관련 치료가 내 몸의 면역력을 올려주는 것이 중요하다.** It is important for pain management treatments to boost my immune system.					
**e. 통증관련 치료가 내 몸의 자가치유 능력을 올려주는 것이 중요하다.** Pain management treatments should enable my body to heal itself.					
**f. 건강은 몸, 마음, 영혼의 조화로 이루어진다.** Health is about harmonizing body, mind, and spirit.					
**g. 삶의 불균형은 질병의 주요 원인이다.** Imbalances in a person's life are a major cause of illness.					
**h. 통증관련 치료는 사람을 전체로 보는 것이 아닌 증상에만 집중해야 한다.** Pain management treatments should concentrate only on symptoms rather than the whole person.					
**i. 통증관련 치료는 사람의 전체적인 안녕(웰빙)을 고려해야 한다.** Pain management treatments should focus on people's overall well-being.					
**j. 나는 내 몸이 스스로 치유할 수 있는 능력이 있다고 믿는다**. I think my body has a natural ability to heal itself.					
**k. 통증관련치료는 몸이 스스로 치유할 수 있는 능력과 상충된다.** There is no need for pain medication to be concerned with natural healing powers.					

통증관련 약에 대한 자세와 믿음
Attitudes and Beliefs about Pain Medications

8. 저희는 통증 조절에 관한 환자분의 입장에 대해 알고자 합니다. 따라서 아래 질문에 대하여 **어느 정도 동의 또는 비동의** 하시는지 선택 부탁드립니다.

We are interested in learning about your attitudes towards treatment of pain. Some of the questions may seem similar to other ones, but please answer all of the questions. For each of the items below, please fill in the number that comes closest to how much you agree with that item.

질문	비동의	약한비동의	모르겠다	약한동의	강한동의
**a. 사람들은 통증관련 약에 쉽게 중독된다.** People get addicted to pain medicine easily.					
**b. 통증이 조금 있을 때부터 진통제를 먹으면, 통증이 심해졌을 때 효과가 없을 수도 있다.** If you take pain medicine when you have some pain, then it might not work as well if the pain becomes worse.					
**c. 진통제를 먹으면 졸린 것이 너무 불편하다.** Drowsiness from pain medication is really a bother.					
**d. 진통제를 먹으면 혼란스러워지는 것이 너무 불편하다.** Confusion from pain medication is really a bother.					
**e. 진통제를 먹으면 생기는 오심이 너무 불편하다.** Nausea from pain medication is really distressing					
**f. 진통제를 먹으면 내가 의도하지 않은, 당황스러운 말을 하게 된다.** Pain medication often makes you say or do embarrassing things					
**g. 진통제를 먹으면 생기는 변비가 너무 불편하다.** Constipation from pain medicine is really upsetting					
**h. 다른 약보다 진통제 부작용은 참을 만하다.** It is easier to put up with pain than with the side effects that come from pain medicine					
**i. 진통제는 별로 통증조절에 도움을 주지 않는다.** Pain medication cannot really control pain.					
**j. 통증에 대해 불평하지 않는 강한 마음을 갖는 것이 중요하다.** It is important to be strong by not talking about pain.					
**k. 의사는 통증조절보다 병 자체를 고치는 것에 집중해야 한다.** It is more important for the doctor to focus on curing illness than to put time into controlling pain.					
**l. 통증이 있다는 것은 병이 점점 나빠진다는 증거이다.** Having pain means the disease is getting worse					
**m. 주사 맞는 것이 싫다.** I do not like having shots.					

Section IV. 한약에 대한 자세와 믿음
Attitudes and Beliefs towards Herbal Medicine

한약은 인기 있는 전통적인 치료 방법 중 하나입니다. 한국에서는 암환자들이 다양한 한약재와 종류(탕, 환, 가루)로 치료를 받습니다. 한약이 오늘날의 의료 시스템에서 부족한 수술, 화학 요법, 방사선과 같은 전통적인 암 치료로 인한 부작용을 완화할 수 있다는 점이 연구에 의해 입증되었습니다. Herbal medicine is one of the most popular traditional treatment methods. In Korea, cancer patients are often treated with a variety of herbal medications in different modes (decoctions, powders, and tablets) to help manage their symptoms, including pain. Herbal medicine has been shown by studies to alleviate side effects from conventional cancer treatments such as surgery, chemotherapy, and radiation.

1. 한약치료에 대한 나의 생각은?

My thoughts in receiving herbal medicine (Decoction, powder, herbal medicine pill etc) treatment is?

질문	비동의	약한비동의	모르겠다	약한동의	강한동의
**a. 한약치료는 과학적 자료에 근거하지 않는다.** herbal medicine treatments are not based on scientific research.					
**b. 내가 받고 있는 기존 치료 (항암, 방사선)를 방해할 수 있다.** herbal medicine treatments may interfere with the conventional cancer treatments.					
**c. 한약치료는 부작용이 있을 수 있다.** herbal medicine treatments may have side effects.					
**d. 한약은 먹기가 또는 준비하기가 불편하다.** herbal medicine may be difficult or unpleasant to prepare or ingest.					
**e. 한약치료는 너무 비싸다.** herbal medicine treatment is too expensive.					
**f. 좋은 한의사 (한약처방)를 찾기가 힘들다.** It is difficult to find good herbal practitioner to prescribe herbs for pain management.					
**g. 한약처방을 받으러 갈 시간이 부족하다.** I don't have enough time to get herbal medicine prescription.					
**h. 한약치료에 대해 잘 모른다.** I don't really know about herbal medicine.					
**i. 한약치료는 내가 가지고 있는 보험이 적용되지 않는다.** My insurance does not cover herbal medicine treatment.					
**j. 한약처방을 받으러 갈 교통이 불편하다.** I don't have transportation to get herbal medicine.					

2. 암 치료관련, 또 다른 건강문제를 해결하기 위해 각자가 가지고 있는 한약에 대한 기대감은 다를 수 있습니다. 한약 사용에 대한 기대감과 관련하여 다음항목에 대해 어느 정도 동의하시나요?

Each individual may have different expectations for using herbs during their cancer treatment and beyond. How much do you agree with the following sentences that describe your expectations for the use of herbs?

	비동의	약한비동의	모르겠다	약한동의	강한동의
**a. 면역력을 올려준다**<br>**Boost my immune system**					
**b. 신체적인 건강을 나아지게 한다**<br>**Improve my physical health**					
**c. 내 증상들을 나아지게 한다**<br>**Reduce my symptoms**					
**d. 암환자로서의 경험하는 것들을 대처하는데 도움을 준다**<br>**Help me cope with the experience of having cancer**					
**e. 생존율을 높인다**<br>**Help me live longer**					
**f. Harmonize my mind and body**<br>**몸과 마음이 조화를 이루도록 도와준다**					
**g. 앞으로의 건강문제가 생기지 않도록 예방해준다**<br>**Prevent future development of health problems**					
**h. Reduce stress**<br>**스트레스를 낮춰준다**					
**i. Help cure my cancer**<br>**암을 치료하도록 도와준다**					

3. 아래질문에 대하여 **어느 정도 동의 또는 비동의** 하시는지 선택 부탁드립니다.

People around us often can influence our decisions to use different types of treatments. Please select the best option to indicate the extent to which you agree or disagree with the following statement

질문	비동의	약한비동의	모르겠다	약한동의	강한동의
**j. 내 가족들은 내가 통증을 위해 한약치료를 받는 것을 권장한다.** My family encourages me to use herbal medicine for pain					
**k. 내 담당의료인(의사, 간호사 등)은 내가 통증을 위해 한약치료를 받는 것을 권장한다.** My health care providers (e.g. doctors, nurses, etc.) encourage me to use herbal medicine for pain.					
**i. 내 담당의료인(의사, 간호사 등)은 내가 통증을 위해 한약치료를 받는 것을 열린 마음으로 받아들여 준다.** My health care providers (e.g. doctors, nurses, etc.) are open to my use of herbal medicine for pain.					
**m. 다른 암 환자들은 내가 통증을 위해 한약치료를 받아야 한다고 생각한다.** Other cancer patients think I should use herbal medicine for pain					
**n. 내 온라인 서포터 그룹은 내가 통증을 위해 한약치료를 받도록 권장한다.** My online support group encourages me to use herbal medicine for pain.					
**o. 내 친구들은 나에게 통증을 위해 한약치료를 받으라고 한다.** My friends ask me to try herbal medicine for pain.					

4. 암 치료 이후 어떤 한약을 사용하셨거나, 사용하고 계시나요?

Which herbs do you use or have you used since your cancer diagnosis?

____

____

____

Section V. 침 치료에 대한 자세와 믿음

Attitudes and beliefs about Acupuncture

침술은 2,500 년 전통의 치료법입니다. 침술은 몸에 특정한 경혈을 통해 에너지의 균형을 맞추고 치유를 촉진하기 위해 머리카락처럼 가는 바늘을 사용합니다. 침술은 만성 관절염이나 요통이 있는 환자들에게 부작용이 거의 없는 안전하고 효과적인 치료법인 것으로 밝혀졌습니다. 침 치료는 보통 6-10 주 동안 매주 방문해야 합니다.
Acupuncture is a 2,500 year-old healing therapy from China. Acupuncture uses hair-thin needles to puncture specific points on the body to balance energy and promote healing. Acupuncture has been found to be a safe and effective treatment with few side effects for patients with chronic arthritis or back pain. A course of acupuncture requires weekly visits for 6-10 weeks.

1. 침 치료의 효과에 대한 기대감은 개인마다 다를 수 있습니다. 만성통증을 치료하는 침 치료에 대한 다음문장들을 읽고, 침 치료 이후의 기대를 설명한 문장에 대해 얼마나 동의하는지 표시해주세요.
Each individual may have different expectations for the effects of acupuncture. If we use the following sentences to describe your expectation of acupuncture's effect on your chronic pain after the entire course of acupuncture therapy, how much do you agree?

	비동의	약한비동의	모르겠다	약한동의	강한동의
**a. 내 만성통증이 많이 나아질 것이다<br>my chronic pain will improve a lot**					
**b. 내 만성통증을 더 잘 대응하게 될 수 있을 것이다<br>I will be able to cope with my chronic pain better**					
**c. 내 만성통증 증상이 나아질 것이다 the symptoms of my chronic pain will disappear**					
**d. 내 몸의 에너지레벨이 증가할 것이다<br>My energy level will increase.**					

2. 만약 침 치료를 받는 것에 대한 장벽이 있다면 어떤 것일까요? 해당되는 부분에 체크 부탁드립니다.
What are some of the barriers on receiving acupuncture for you?

질문	비동의	약한비동의	모르겠다	약한동의	강한동의
**a. 침 치료는 과학적 자료에 근거하지 않는다.** Acupuncture treatments are not based on scientific research.					
**b. 내가 받고 있는 기존치료(항암,방사선)를 방해할 수 있다.** It may interfere with the conventional cancer treatments.					
**c. 침 치료는 부작용이 있을 수 있다.** Acupuncture treatments may have side effects.					
**d. 침 치료는 아프다.** Acupuncture needling is too painful.					
**e. 침 치료는 너무 비싸다.** Acupuncture treatments cost too much money.					
**f. 좋은 한의사를 찾기가 힘들다.** It is hard to find good acupuncturists.					
**g. 침 치료를 받으러 갈 시간이 부족하다.** I don't have knowledge about acupuncture treatments.					
**h. 침 치료에 대해 잘 모른다.** I don't have transportation to acupuncture treatments.					
**i. 내 보험은 침 치료가 커버되지 않는다.** acupuncture treatments are not covered by my insurance.					
**j. 침 치료를 받으러 가는 교통이 너무 불편하다.** I don't have transportation to acupuncture treatments.					

3. 우리 주변에 있는 사람들은 종종 우리의 치료방법 선택에 대해 영향을 미치곤 합니다. 아래질문에 대하여 **어느 정도 동의 또는 비동의** 하시는지 선택 부탁드립니다.

People around us often can influence our decisions to use different types of treatments. Please select the best option to indicate the extent to which you agree or disagree with the following statements.

질문	비동의	약한비동의	모르겠다	약한동의	강한동의
**a. 내 가족들은 내가 통증을 위해 침 치료를 받는 것을 권장한다.** My family encourages me to use acupuncture.					
**b. 내 담당의료인(의사, 간호사 등)은 내가 통증을 위해 침 치료를 받는 것을 권장한다.** My health care providers (e.g. doctors, nurses, etc.) encourage me to use acupuncture.					
**c. 내 담당의료인(의사, 간호사 등)은 내가 통증을 위해 침 치료를 받는 것을 열린 마음으로 받아들여 준다.** My health care providers (e.g. doctors, nurses, etc.) are open to my use of acupuncture.					
**d. 다른 암 환자들은 내가 통증을 위해 침 치료를 받아야 한다고 생각한다**. Other cancer patients think I should use acupuncture.					
**e. 내 온라인 서포터 그룹은 내가 통증을 위해 침 치료를 받도록 권장한다**. My online support group encourages me to use acupuncture.					
**f. 내 친구들은 나에게 통증을 위해 침 치료를 받으라고 한다.** My friends ask me to try acupuncture.					

Section VI. 선호하는 치료

1. 전반적으로, 통증치료에 다음 항목을 **사용하는 것에 대한 의지를** 선택해주세요.
Overall, how willing or unwilling are you to **use the following therapies** to manage your pain?

	절대 사용하고 싶지 않다	별로 사용하고 싶지 않다	잘 모르겠다	조금 사용하고 싶다	매우 사용하고 싶다
**a. 진통제** Pain medications					
**b. 침 치료** Acupuncture					
**c. 한약치료** Herbal medicine					

2. 통증관리에 어떤 방법을 쓰는 것을 선호하시는지 순서를 매겨주세요.

Please indicate your preferences among the pain treatments below, from your first choice (the treatment you prefer MOST) to your fourth choice (the one you LEAST prefer).

	First choice 가장 선호함	Second choice 다음으로 선호함	Third choice 가장 덜 선호함
Pain medication 진통제	○	○	○
Acupuncture 침 치료	○	○	○
Herbal medicine 한약치료	○	○	○

3. 암환자의 웰빙 (안녕)을 위해서 필요한 도움에 대해 나누어주실 정보가 있다면 기재 부탁드립니다. 통증을 위한 치료를 받는데 가장 힘든 점이 무엇인가요? 당신과 같은 환자들을 도와주려면 어떤 것이 필요할까요?

Please share any information that could help us support the well-being of patients with cancer. What challenges do you face in getting treatments for pain? What might help patients like you?

____

____

____

____

____

____

____

____

____

작성날짜 date of completion: 2023 년 ____월 _____일

수련의 서명 signature of doctor (문항확인): ___________ (인)

작성자 서명 signature of patient :___________ (인)

설문에 응해 주셔서 대단히 감사합니다.

Thank you very much for taking the time to complete this survey!
